# Comprehensive analysis of mutational signatures reveals distinct patterns and molecular processes across 27 pediatric cancers

**DOI:** 10.1038/s43018-022-00509-4

**Published:** 2023-01-26

**Authors:** Venu Thatikonda, S. M. Ashiqul Islam, Robert J. Autry, Barbara C. Jones, Susanne N. Gröbner, Gregor Warsow, Barbara Hutter, Daniel Huebschmann, Stefan Fröhling, Marcel Kool, Mirjam Blattner-Johnson, David T. W. Jones, Ludmil B. Alexandrov, Stefan M. Pfister, Natalie Jäger

**Affiliations:** 1 Hopp Children’s Cancer Center Heidelberg (KiTZ), Heidelberg, Germany; 2 Division of Pediatric Neurooncology, German Cancer Research Center (DKFZ), Heidelberg, Germany; 3 German Cancer Consortium (DKTK), Heidelberg, Germany; 4 Department of Cellular and Molecular Medicine and Department of Bioengineering, Moores Cancer Center, UC San Diego, La Jolla, CA USA; 5 Department of Pediatric Oncology, Hematology and Immunology, Heidelberg University Hospital, Heidelberg, Germany; 6 Pediatric Glioma Research Group, DKFZ, Heidelberg, Germany; 7 Omics IT and Data Management Core Facility (W610), DKFZ, Heidelberg, Germany; 8 Computational Oncology Group, Molecular Precision Oncology Program, National Center for Tumor Diseases (NCT) Heidelberg, DKFZ, Heidelberg, Germany; 9 Division of Applied Bioinformatics, DKFZ, Heidelberg, Germany; 10 Pattern Recognition and Digital Medicine, Heidelberg Institute for Stem Cell Technology and Experimental Medicine (HI-STEM), Heidelberg, Germany; 11 Division of Translational Medical Oncology, NCT Heidelberg and DKFZ, Heidelberg, Germany; 12 Princess Máxima Center for Pediatric Oncology, Utrecht, the Netherlands; 13 Present Address: Global Computational Biology and Digital Sciences, Boehringer Ingelheim RCV GmbH, Vienna, Austria

**Keywords:** Paediatric cancer, Functional clustering, Cancer, Cancer genomics

## Abstract

Analysis of mutational signatures can reveal underlying molecular mechanisms of the processes that have imprinted the somatic mutations found in cancer genomes. Here, we analyze single base substitutions and small insertions and deletions in pediatric cancers encompassing 785 whole-genome sequenced tumors from 27 molecularly defined cancer subtypes. We identified only a small number of mutational signatures active in pediatric cancers, compared with previously analyzed adult cancers. Further, we report a significant difference in the proportion of pediatric tumors showing homologous recombination repair defect signatures compared with previous analyses. In pediatric leukemias, we identified an indel signature, not previously reported, characterized by long insertions in nonrepeat regions, affecting mainly intronic and intergenic regions, but also exons of known cancer genes. We provide a systematic overview of COSMIC v.3 mutational signatures active across pediatric cancers, which is highly relevant for understanding tumor biology and enabling future research in defining biomarkers of treatment response.

## Abstract (web summary)

Jäger and colleagues analyze single-base substitution and indel mutational signatures across 27 pediatric cancer types, revealing marked differences in mutational patterns compared with adult cancers and insights into underlying molecular mechanisms.

## Main

Childhood cancers have a much lower incidence rate when compared with the overall incidence of cancers in adulthood. Nevertheless, cancer remains one of the leading causes of death by disease among children^1^. Growing research evidence suggests that childhood cancers are notably different in terms of molecular features and therapy response when compared with their adult counterparts. Most prominently, recent pan-cancer analyses revealed that pediatric cancers show a substantially lower mutation burden in contrast to common adult cancers^2,3^. However, knowledge of the underlying mutational processes that contribute to the somatic mutation burden and tumor development in pediatric cancer is still limited.

Somatic mutations including single base substitutions (SBS), small insertions and deletions (indels or IDs), copy number changes and other genomic rearrangements can, in principle, be caused solely by endogenous processes (for example, defects in DNA repair, errors in DNA replication, damage due to reactive oxygen species, and so on), or by the additional influence of exogenous causes such as exposure to ultraviolet light, tobacco smoking and numerous other factors^4^. Previous pan-cancer analyses, focused mainly on adult tumor types, employed mathematical models based on non-negative matrix factorization (NMF) to identify patterns of somatic mutations, termed mutational signatures, and used these patterns to infer underlying mutational processes^5^. Since then, mutational signatures have been used to understand tumor development, to identify gene alterations associated with mutational processes and, importantly, as biomarkers for predicting treatment response^6–12^. A total of more than 40 SBS signatures and 17 small ID signatures were identified in a recent analysis as part of the pan-cancer analysis of whole genomes (PCAWG) consortium^13,14^, updating and expanding the previous set of reference mutational signatures (that is, mutational signatures in COSMIC v.2).

Previous studies of individual pediatric tumor types and pan-cancer approaches analyzed mutational signatures as part of molecular tumor landscape analyses using the COSMIC v.2 reference signatures^15–20^. Here, we carried out an extensive analysis encompassing mutational signatures of SBS and indels in 785 whole-genome sequenced pediatric tumor-normal pairs. These were subsequently compared with COSMIC v.3 (ref. ^14^) signatures to identify overlap with the latest set of known mutational signatures. This study establishes the analysis of SBS and ID mutational signatures in one of the largest pediatric pan-cancer cohorts based on whole-genome sequencing (WGS) to date.

## Results

### Somatic mutation frequencies across 27 pediatric cancers

**Fig. 1:**
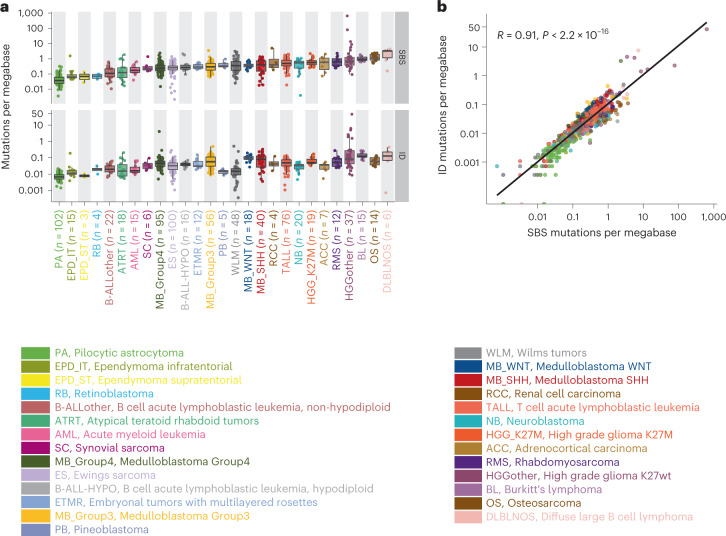
Mutation burden of SBS and small IDs across 27 pediatric cancer types. **a**, SBS and small ID mutation burden of the PPC-WGS cohort. The numbers of samples for each tumor type are shown next to the labels. Each dot represents one tumor sample. Tumor types are ordered by the median numbers of SBS. Each boxplot shows lower and upper quartile, and median line is indicated. Range of whiskers: 1.5× interquartile range. **b**, Correlation between SBS and ID mutations per megabase across the cohort (Spearman’s correlation, confidence interval 0.95. *P* value is indicated; *n* = 785 tumors). Source data

**Extended Data Fig. 1:**
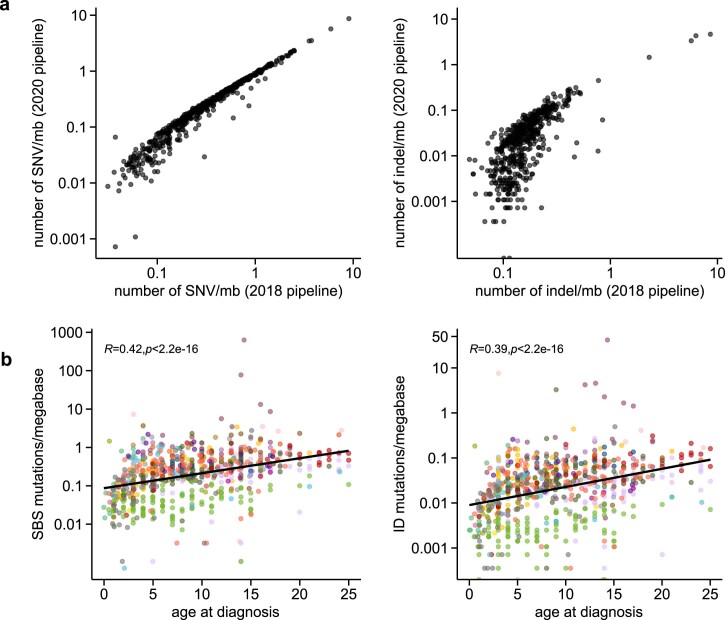
Correlation of SBS and ID mutations with age at tumor diagnosis. a) Comparison of number of mutation calls identified between 2018 (in Gröbner et al., 2018) and the current consensus pipeline (includes updated DKFZ in-house pipeline and mutect2, n = 537 tumors) b) Correlation of SBS and ID mutations with age across the PPC-WGS cohort (Spearman’s correlation, confidence interval 0.95. P value is indicated, n = 729 tumors). Source data

A dataset of 785 whole-genome sequenced tumor-normal pairs was compiled from our previously published study^2^ (the PedPanCan cohort, PPC-WGS, https://www.kitz-heidelberg.de/en/research/datacommons/pedpancan) and the St. Jude pediatric cancer genome project (https://www.stjude.org/research/translational-innovation/pediatric-cancer-genome-project.html), spanning, in total, 27 molecularly defined entities of childhood cancer (Fig. 1a). SBS and IDs were identified using a consensus variant-calling pipeline, which includes an updated German Cancer Research Center (DKFZ) in-house mutation calling pipeline and mutect2 (ref. ^21^), substantially improving the quality of somatic variants identified (Extended Data Fig. 1a). In total, across the cohort we identified 2,834,456 SBS and 252,722 IDs. The number of SBS mutations per megabase (median 0.2350; range 0.0007–625.5) and IDs (median 0.0036; range 0.00036–41.43) was highly variable both across individual tumors and across different cancer types (Fig. 1a and Supplementary Table 1). The lowest overall somatic mutation burden was observed in pilocytic astrocytoma (median 0.034 total mutations per megabase) and the highest in DLBLNOS (diffuse large B cell lymphoma, not otherwise specified, median 1.83 total mutation per megabase). Although the indel mutation burden per tumor was low, numbers of SBS and IDs were significantly correlated across tumors (Spearman *r* = 0.91; Fig. 1b). As previously described in different cancer types, the mutation burden of both SBS and IDs is also clearly correlated with age in this pediatric cohort (Extended Data Fig. 1b). A small number (*n* = 3) of tumors classified as high-grade glioma (HGG, K27wt) with germline alterations in DNA mismatch repair genes^22–25^ (*MSH6*, *PMS2*) showed a hypermutator phenotype^2^, with a median of 79.23 total mutations per megabase (Fig. 1b).

### Mutational signatures in pediatric cancers

**Fig. 2:**
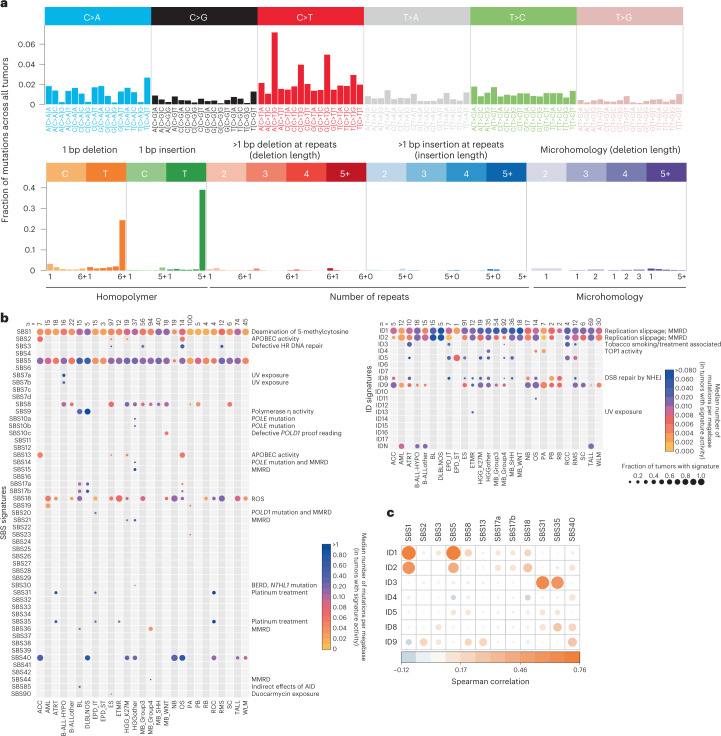
SBS and ID signature activity across pediatric cancers. **a**, Example profiles of SBS and ID profiles used for mutational signature extraction. These profiles were generated using PPC-WGS (*n* = 785 tumors) cohort averages. **b**, Number of mutations contributed by each mutational signature to the PPC-WGS tumors. Circle size indicates fraction of tumors with signature activity in a cancer type and color indicates the median number of mutations per megabase due to a signature in a specific cancer type; AID, activation-induced cytidine deaminase; BERD, base excision repair deficiency; MMRD, mismatch repair deficiency; *n*, number of tumors in each cancer type with at least 20 mutations. **c**, Correlation between SBS and ID signature activities (Spearman correlation, confidence interval 0.95). Source data

To extract mutational signatures active in this pediatric pan-cancer cohort, we generated 96-context SBS and 83-context ID mutational catalogs (Fig. 2a) and used SigProfiler^14,26^ and SignatureAnalyzer^14,27,28^ (hereafter SigAnalyzer), two extensively used methods based on different principles of NMF. In addition, a third non-NMF based method called hierarchical Dirichlet process^29,30^ (HDP) was implemented for leukemias to identify ID signatures. Due to the very low number of double base substitutions (DBS) per pediatric tumor, we extracted signatures only from SBS and IDs. Since SigProfiler reference signatures are used widely in the scientific community (COSMIC v.3), results of SigProfiler are outlined here and SigAnalyzer results are presented in Supplementary Tables 2, 3, 4 and 5.

### SBS signatures

**Extended Data Fig. 2:**
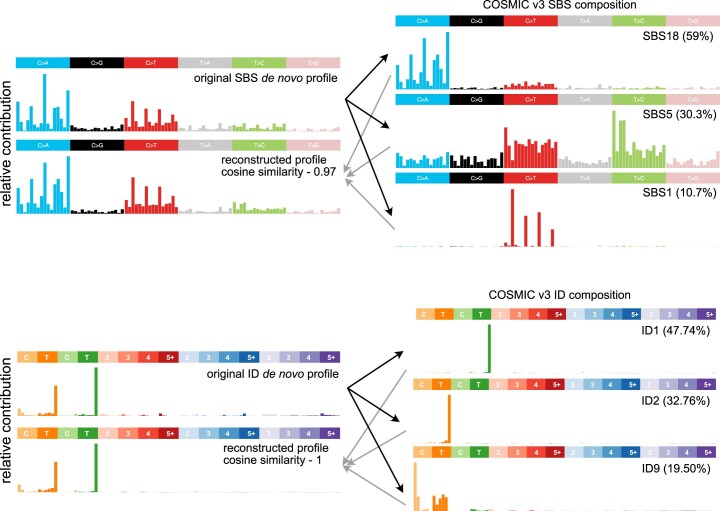
Example decomposition of SBS and ID de novo profiles into known COSMIC v3 signatures. Example decomposition of SBS and ID *de novo* profiles into known COSMIC v3 signatures. Source data

**Extended Data Fig. 3:**
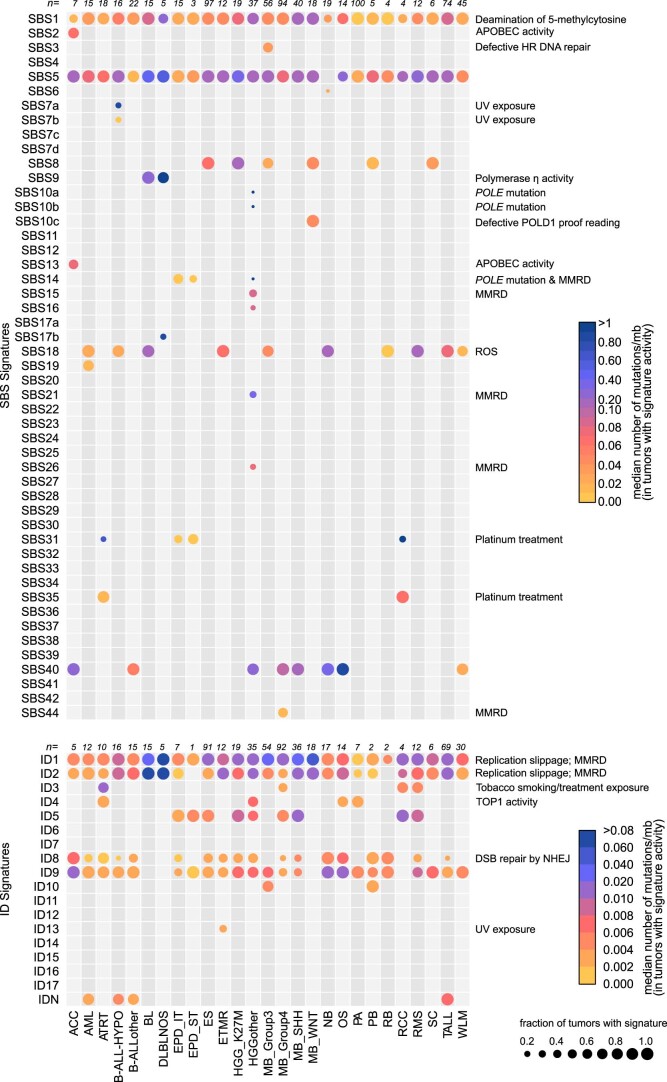
SBS and ID signature activities in pediatric tumors extracted by SigAnalyzer. Similar to Fig. 2b, color of the circle indicates the median number of mutations/megabase due to a signature and size of the circle indicates the fraction of tumors showing the signature in a cancer type. ‘n’ in the figure indicates the number of tumors in each cancer type with at least 20 mutations. Source data

**Extended Data Fig. 4:**
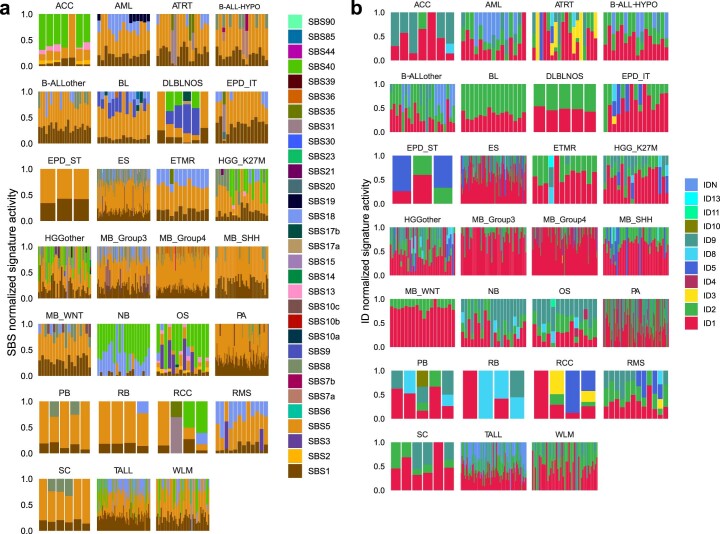
Normalized SBS and ID signatures in each individual tumor according to SigProfiler. Normalized SBS and ID signatures in each individual tumor according to SigProfiler. Source data

**Extended Data Fig. 5:**
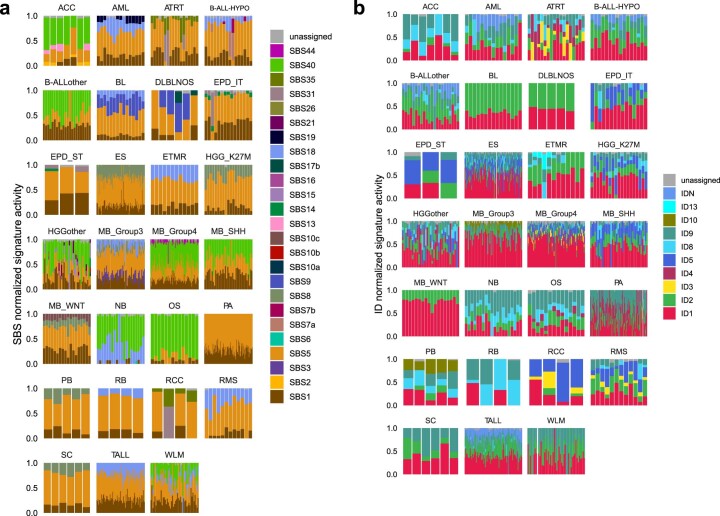
Normalized SBS and ID signatures in each individual tumor according to SigAnalyzer. Normalized SBS and ID signatures in each individual tumor according to SigAnalyzer. Source data

**Extended Data Fig. 6:**
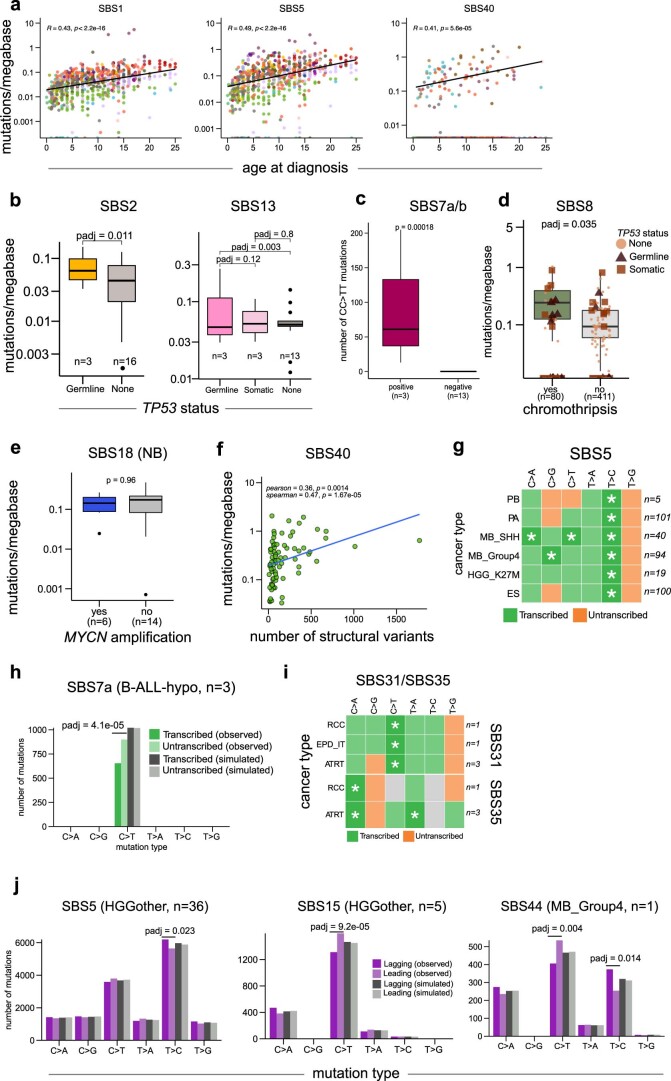
Association of SBS signatures with patient age and genomic features. a) Correlation of age at diagnosis and SBS signature activity. Colors indicate different cancer types as in Fig. 1a (Spearman’s correlation, confidence interval 0.95. P value is indicated, n = 727 tumors) b) Association of *TP53* mutation status and SBS2, SBS13 signature activities c) Number CC > TT dinucleotide substitutions in SBS7a/b positive B-ALL-HYPO tumors d) Association of SBS8 and chromothripsis e) Association of SBS18 and *MYCN* amplification in neuroblastoma b-e: boxplots show lower and upper quartile and median line is indicated. Range of whiskers: 1.5 x interquartile range (significance values are computed with two-sided Wilcoxon rank sum test, confidence interval 0.95. Wherever appropriate, p-values are adjusted for multiple testing with Benjamini-Hochberg method). The ‘n’ in b-e represents the number of tumors f) Association of SBS40 with genomic instability. Colors are same as SBS signature color coding as in Extended Data Fig. 4, 5 (Spearman’s and Pearson’s correlation, confidence interval 0.95, n = 75 tumors) g-i) Transcriptional strand asymmetries of SBS5, SBS7a, SBS31 and SBS35 (Fischer exact test, p.values are adjusted for multiple testing with Benjamini-Hochberg method) j) Replication strand asymmetries of SBS5, SBS15, SBS44 (Fischer exact test, p.values are adjusted for multiple testing with Benjamini-Hochberg method). The ‘n’ in figures g-j indicates the number of tumors used for analysis. Source data

In total, 29 SBS signatures were extracted that matched with COSMIC v.3 SBS signatures and present in at least one tumor type of this cohort (Fig. 2b, Supplementary Table 6 and Extended Data Fig. 2; Methods). The mutational signatures identified by SigProfiler, which affected a large fraction of tumors per entity, were also consistently identified with SigAnalyzer (Extended Data Figs. 3, 4 and 5). Amongst these, SBS1 and SBS5 were present in 98.2% and 96.6% of samples across the cohort, respectively (Fig. 2b and Supplementary Tables 6 and 7). As described in adult cancers^5,7^, and for a small fraction of pediatric brain tumors^2^, the clock-like nature of SBS1 and SBS5 was also observed in this cohort, with a significant correlation of signature activity with age at diagnosis^7^ (Extended Data Fig. 6a). An additional signature with unknown etiology, namely SBS40, was found to be active in nine pediatric cancer types and 11.7% of tumors across the cohort (Fig. 2b and Supplementary Table 7) and was also correlated with age at diagnosis (Extended Data Fig. 6a), suggesting an additional clock-like signature.

SBS2 and SBS13 were reported to be due to the activity of APOBEC (apolipoprotein B mRNA editing enzyme, catalytic polypeptide) enzymes^14^ and in this cohort they were found to be present only in adrenocortical carcinoma (ACC), Ewing sarcoma (ES), high-grade glioma (HGG, K27M) and osteosarcoma (OS) (Fig. 2b and Supplementary Table 6). In a cross-cohort comparison, mutations attributed to SBS2 and SBS13 were significantly higher in *TP53* mutated compared with wildtype tumors (Extended Data Fig. 6b). This observation is in line with previous studies, which identified a link between p53 loss and elevated *APOBEC3B* expression^31,32^. However, not all *TP53* germline mutated tumors in this cohort showed SBS2 or SBS13 activity, including the *TP53*-defined subtype of SHH-medulloblastoma (see Supplementary Table 8 for an overview of tumors with *TP53* mutations), suggesting tissue-specific association of *TP53* mutations and APOBEC signature activities.

Ultraviolet light (UV) exposure signatures SBS7a/b^14^ were identified exclusively in hypodiploid B cell acute lymphoblastic leukemia (B-ALL-HYPO) (Fig. 2b and Supplementary Table 6). In general, UV-light-affected tumors show an enrichment of dipyrimidine substitution mutations (CC>TT). Consistently, SBS7a/b positive tumors show a significantly higher number of CC>TT doublet base substitutions when compared with SBS7a/b-negative tumors in the B-ALL-HYPO subtype (Extended Data Fig. 6c). Although multiple independent studies have recently identified signatures apparently linked with UV light exposure in pediatric B-ALL^3,33^, the exact mechanism of how this contributes to leukemogenesis, and whether UV light is really the cause or whether this signature is rather mimicked by another process, remains to be fully elucidated.

Additional relatively frequent signatures across cancer types included SBS8 and SBS18, present in 20.17% and 27.17% of our cohort, respectively (Fig. 2b and Supplementary Table 7). Signature8 from COSMIC v.2 was proposed to be due to homologous recombination (HR) repair pathway gene mutations^2^. We did not identify any such mutations in SBS8-positive pediatric tumors in this analysis. Recent evidence suggests that the SBS8 signature is due to DNA damage caused by late replication errors^34^.

SBS18 is the result of DNA damage caused by reactive oxygen species (ROS)^14^ and its activity was observed in 17 out of 27 cancer types analyzed in this cohort (Fig. 2b). Among these 17 cancer types, the highest fractions of tumors with SBS18 signature activity were found in neuroblastoma (90%, NB) and rhabdomyosarcoma (83.3%, RMS) (Supplementary Table 7).

Mutations attributed to signature SBS9 are due to DNA damage caused by polymerase η activity and were observed only in Burkitt lymphoma (BL) and diffuse large B cell lymphoma (DLBLNOS) in this cohort. SBS9 activity has been found previously to be enriched in tumors with immunoglobulin gene hypermutations^35^. Although we identified increased activity of SBS9 in immunoglobulin heavy chain (IGHV)-mutated tumors (0.36 mutations per megabase), we did not observe a significant difference compared with IGHV wildtype tumors (0.15 mutations per megabase) in this cohort. Signature 10 from COSMIC v.2 has been split into SBS10a and SBS10b in the COSMIC v.3 signatures^14^. Mutations from high-grade glioma hypermutator tumors were attributed to both SBS10 signatures (polymerase ε mutations), as well as SBS14 and SBS15 (defective DNA mismatch repair; Fig. 2b).

We also identified infrequent signatures such as SBS21 and SBS30 in pediatric high-grade glioma, which are the result of defective DNA mismatch repair, SBS36 in Burkitt lymphoma and Group4 medulloblastoma, which is the result of defective base excision repair, and SBS44 in Group4 medulloblastoma, also caused by defective DNA mismatch repair. For these signatures, we have not identified any associated consistent genetic alteration enriched across tumors. However, a few individual tumors harbored nonsynonymous somatic or germline mutations in different genes involved in DNA mismatch repair and nucleotide excision repair pathways (for example, *TP53*, *MLH1*; Supplementary Table 9).

Next, we sought to identify any associations between SBS signature activity, chromothripsis, genomic instability and strand asymmetry of identified signatures. These hypothesis-driven association analyses were based mainly on previous evidence and findings in our 2018 pediatric pan-cancer study^2^. In a cross-cohort analysis, mutations attributed to SBS8 were significantly higher in chromothripsis positive tumors (Extended Data Fig. 6d and Supplementary Table 8). Signature SBS18 was first described in neuroblastoma^5^ and attributed to ROS^36^. Amplification of *MYCN* enhances glutaminolysis in neural crest progenitor cells, which in turn induces oxidative stress by ROS production. In a recent study of neuroblastoma^37^, SBS18 prevailed in *MYCN*-amplified tumors in agreement with previous literature. However, we did not observe this association (Extended Data Fig. 6e), which might be explained by the low number of neuroblastomas in our cohort (*n* = 20 samples, of which 6 are *MYCN*-amplified). Genomic instability, quantified here as the total number of structural variants (deletions, duplications, inversions and translocations) identified in a tumor, was highly correlated with SBS40 in cross-cohort analysis (Extended Data Fig. 6f). This observation may indicate that mechanisms involved in genomic rearrangements contribute to mutational processes underlying SBS40. In adult cancer types, several signatures have been reported to exhibit strand asymmetry^35^. Here, we observed transcriptional strand asymmetry for SBS5, that is, an enrichment of T>C mutations on the transcribed strand in different cancer types (Extended Data Fig. 6g). Similarly, UV signature SBS7a (identified in B-ALL-HYPO) showed an enrichment of C>T mutations on the untranscribed strand (Extended Data Fig. 6h). Two platinum-treatment-related signatures, SBS31 and SBS35, have also been observed to exhibit strand asymmetry at C>T and C>A transcribed strands, respectively (Extended Data Fig. 6i). Similarly, replication strand asymmetry has been observed for SBS5 with an enrichment of lagging strand mutations at T>C, for SBS15 with an enrichment of leading strand at C>T in high-grade glioma tumors and for SBS44 with an enrichment of leading strand mutations at C>T and lagging strand mutations at T>C in Group4 medulloblastoma (Extended Data Fig. 6j).

### ID signatures

A total of ten ID signatures were identified from our cohort, including nine that were overlapping with COSMIC v.3 signatures and a signature we identified here, which we termed IDN (Fig. 2b and Supplementary Table 10). Similar to SBS signatures, most frequent ID signatures from the SigProfiler de novo extraction were consistently identified with SigAnalyzer as well (Extended Data Figs. 3, 4 and 5). As observed in the PCAWG mutational signature analysis, ID1, ID2, ID5, ID8 and ID9 were active across tumors of multiple cancer types (Fig. 2b, Extended Data Fig. 3 and Supplementary Table 10)^14^. ID1 and ID2, which are the result of DNA damage induced by replication slippage, were present in around 92.2% and around 67.2% of tumors in this cohort, respectively (Supplementary Table 11; the most prevalent ID signatures in this cohort). Although ID5 and ID9 were present in multiple cancer types, mutations were attributed to these signatures in a relatively small percentage of tumors (Supplementary Table 11). ID8, a signature caused by the potential damage induced by DNA double-strand break (DSB) repair by nonhomologous end joining (NHEJ) was present in 4.8% (*n* = 38) of the whole cohort. The large cohort analysis of tumors as part of PCAWG revealed that ID8 activity was correlated with age at diagnosis, suggesting a clock-like behavior of this signature^14^. However, we did not observe such a correlation in our cohort, potentially indicating different rates of ID8 mutations in pediatric tissues.

**Extended Data Fig. 7:**
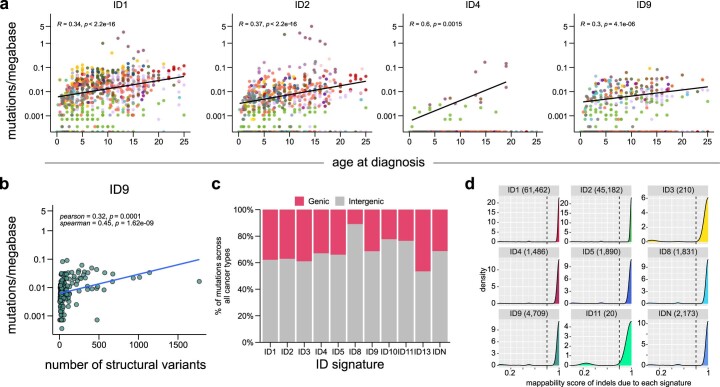
Association of ID signatures with patient age and genomic features. a) Correlation of age at diagnosis and ID signature activity. Colors indicate different cancer types as in Fig. 1a (Spearman’s correlation, confidence interval 0.95. P value is indicated, n = 727 tumors) b) Association of ID9 and genomic instability. Color indicates the ID signature color as in Extended Data Figs. 4, 5 (Spearman and Pearson correlation, confidence interval 0.95, n = 155 tumors) c) Distribution of ID signature mutations in genic and intergenic regions d) Distribution of mappability scores for indel mutations due to each ID signature. Total number of mutations due to each ID signature is indicated in the plot. Source data

Next, we sought to identify potential associations of indel signature activity with age, *TP53* mutation status, chromothripsis and genomic instability. Of the ID signatures active in our cohort, we observed that ID1, ID2 and ID9 signature activity is highly correlated with age at diagnosis (Extended Data Fig. 7a), revealing their clock-like behavior in pediatric cancers as observed in their adult counterparts^14^. Although only present in three cancer types and a small fraction of tumors (3.2% of the cohort, *n* = 25 tumors, Supplementary Tables 10 and 11), a significant correlation between ID4 activity and age at diagnosis has been observed (Extended Data Fig. 7a). A recent study suggested that the ID4 mutational pattern is potentially due to topoisomerase1 activity and that such a pattern could be present both in somatic cancer cells and the germline^38^. The total number of structural variants was highly correlated with ID9 signature activity across the cohort (Extended Data Fig. 7b), suggesting the contribution of mechanisms involved in genomic instability to ID9 mutations. In addition, rather rare signatures in adult tumors (that is, ID3, ID10, ID11 and ID13) were also observed in a small number of tumors in our cohort (Supplementary Tables 10 and 11). However, we did not identify any common genomic alterations across these tumors. Due to the very low number of indels in our cohort, we could not perform a systematic analysis of strand asymmetry across identified signatures. However, similar to results reported in a recent study^39^ based on adult cancers, we observed an enrichment of mutations attributed to different signatures in intergenic regions (Extended Data Fig. 7c). Of note, assessing the mappability of indels assigned to each of the ID signatures revealed that a large fraction of indels were identified from uniquely mapping genome regions (Extended Data Fig. 7d).

Finally, we performed a correlation analysis of SBS and ID signature activities across the cohort to understand whether any of the SBS and ID signatures co-occurred. We observed a high correlation among clock-like signatures SBS1, SBS5 and ID1, ID2 (Fig. 2c). In addition, the ID3 signature was highly correlated with SBS31 and SBS35, both of which are due to DNA damage caused by platinum treatment (Fig. 2b,c). Although ID3 has been hypothesized to be associated with tobacco smoking, in our cohort we observed ID3 co-occurrence with SBS31 and SBS35, suggesting possible DNA damage due to treatment or additional mechanisms generating similar mutational patterns in pediatric tumors.

### ID signatures in pediatric leukemias

**Fig. 3:**
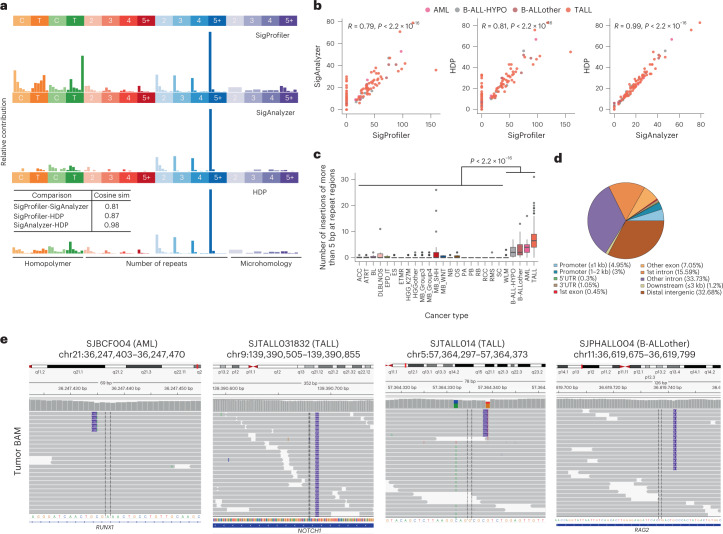
The IDN indel mutational signature in pediatric leukemias. **a**, Profiles of IDN extracted by SigProfiler, SigAnalyzer and HDP. Text box in lower panel indicates cosine similarity comparison across three methods. **b**, Correlation of activities of IDN as derived by three methods from **a** (Spearman’s correlation, confidence intervals 0.95. *P* value is indicated; *n* = 129 samples belonging to leukemias). **c**, Comparison of IDN feature mutations across different cancer types. Each boxplot shows lower and upper quartile and median line is indicated. Range of whiskers: 1.5× interquartile range (two-sided Wilcoxon rank sum test, confidence interval 0.95. *P* value is indicated; number of samples for each cancer type are same as Fig. 1a). **d**, Distribution of IDN feature mutations in the genome. UTR, untranslated region. **e**, IGV screenshots of IDN feature mutations in representative samples. Source data

The indel signature we termed IDN, not yet reported elsewhere, was identified exclusively in different types of pediatric leukemia. The features of IDN have not been reported previously in COSMIC v.3. This signature has been identified consistently by three independent methods with high similarities (Fig. 3a and Supplementary Table 12). This indel signature is characterized by elevated insertions of more than 1 base pair (bp) at nonrepeat regions, often with insertions more than 5 bp in length (Fig. 3a). The number of mutations attributed to this signature by three different de novo signature extraction methods were also highly correlated (Fig. 3b). Next, we compared the total number of the signature feature mutations across different cancer types and identified a significant enrichment in B-ALL, AML and T-ALL (Fig. 3c), with the highest fraction of the IDN signature in T-ALL, but not specifically enriched in any subtype of T-ALL or any underlying somatic alteration. Analysis of the genomic distribution of these mutations suggests that a large fraction of these insertions are present in intronic and distal intergenic regions, but also in coding exons, including insertions in known cancer genes, like *NOTCH1* (Fig. 3d,e) and *PHF6*. To ensure that mutations attributed to the IDN signature were not due to variant-calling artifacts, we visually inspected individual mutations in Integrative Genomics Viewer (IGV) and confirmed their high quality, for example, their occurrence on a single allele when neighboring heterozygous single-nucleotide polymorphisms (SNPs) were in close proximity, spanned by the same reads (Fig. 3e; SJTALL014). Next, we performed a correlation analysis of IDN activity with age at diagnosis and other SBS signatures in these tumors. Although we did not identify a significant correlation with age (Spearman *r* = *0*.26, false discovery rate (FDR) = 0.072; Pearson *r* = 0.22, FDR = 0.052), we observed strong correlation with SBS1 (Spearman *r* = 0.77, FDR = 3.7 × 10^–11^; Pearson *r* = 0.56, FDR = 1.9 × 10^–8^) and SBS40 (Spearman *r* = 0.79, FDR = 0.009; Pearson *r* = 0.38, FDR = 0.0005) (Supplementary Table 13).

In summary, we extracted 29 SBS and ten ID signatures, including the newly identified IDN signature, in our pediatric pan-cancer cohort. The total number of signatures is lower than those identified in adult cancers^14^ and a large fraction of mutations (41.7% and 42.8% of SBS mutations; 87% and 100% of ID mutations in nonhypermutated and hypermutated samples, respectively) were attributed to clock-like signatures such as SBS1, SBS5, ID1 and ID2. A significant difference in terms of mutations attributed to APOBEC signatures (SBS2 and SBS13) has been observed depending on *TP53* mutation status and SBS8 activities based on chromothripsis.

### HR repair defect signature activities

The HR repair pathway is an error-free mechanism to repair DNA DSBs^40^. Genomic alterations in components of the HR pathway, mainly in *BRCA1/2*, lead to a characteristic pattern of SBSs and larger deletions in microhomology (MH) regions—a phenotype also termed BRCAness^41^. Previous studies focusing mainly on breast and ovarian cancers have identified a strong correlation between *BRCA1/2* biallelic pathogenic mutations and activity of signature.3 (COSMIC v.2). However, a substantial fraction of these tumors showed signature.3 activity without any identifiable alterations in HR pathway components^5,11,14,42^. Discerning the activity of HR defect COSMIC v.3 signatures SBS3 and ID6 in tumors is important as it has been shown previously to be associated with the therapeutic response to platinum and poly ADP-ribose polymerase (PARP) inhibitor treatment^12,41–45^.

**Fig. 4:**
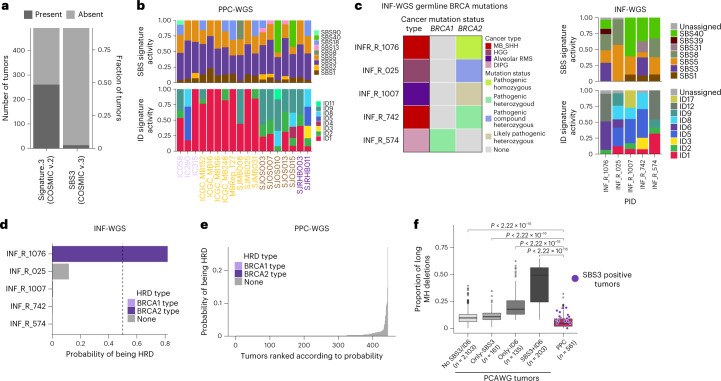
HR defect signatures activity in pediatric cancers. **a**, Comparison of the number of tumors with Signature.3 (COSMIC v.2) and SBS3 (COSMIC v.3) in matched samples from Gröbner et al.^2^ and current study (*n* = 537 matching WGS tumors). **b**, Normalized SBS and ID signature activities in tumors with SBS3 signature activity **c**, *BRCA1/2* mutation status of five INFORM whole-genome-sequenced tumors (left) and their SBS and ID signature activities (right). MB_SHH, Sonic hedgehog subgroup of medulloblastoma; HGG, high-grade glioma; DIPG, diffuse intrinsic pontine glioma; RMS, rhabdomyosarcoma. **d**, Probabilities of being HRD for *n* = 5 INF-WGS tumors. **e**, Probabilities of tumors in *n* = 599 PPC-WGS tumors of being HRD. **f**, Comparison of MH deletion proportion between PCAWG and PPC-WGS cohorts. Each boxplot shows lower and upper quartile and median line is indicated. Range of whiskers: 1.5× interquartile range (two-sided Wilcoxon rank sum test, confidence interval 0.95; *P* value is indicated). Source data

**Extended Data Fig. 8:**
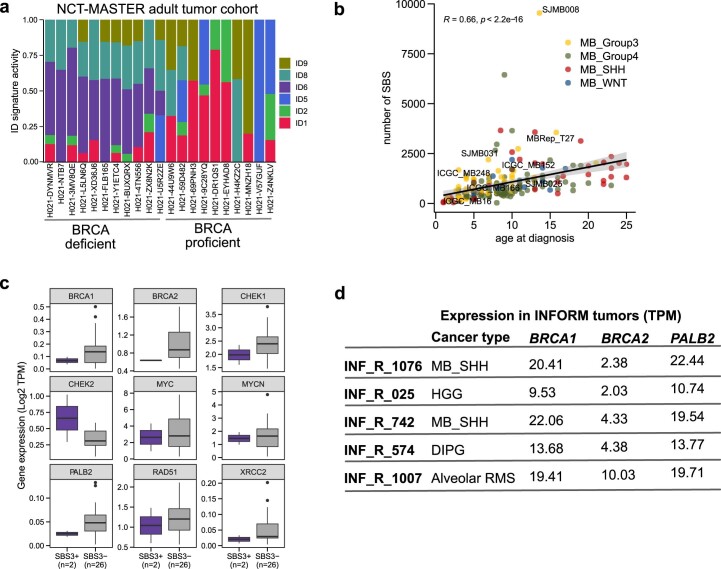
ID signatures in adult cohort and analysis of SBS3 positive tumors. a) ID signature activities in adult tumors of NCT-MASTER (n = 21 tumors) b) Correlation of SBS mutation burden and age at diagnosis in medulloblastoma (Spearman’s correlation, confidence interval 0.95. P value is indicated, n = 208 tumors) c) HR pathway gene expression difference between SBS3 positive and negative tumors of Group3 subgroup of medulloblastoma. Boxplots show lower and upper quartile and median line is indicated. Range of whiskers: 1.5 x interquartile range. ‘n’ in the figure indicates the number of tumors d) HR pathway gene expression in INFORM whole-genome sequenced tumors. Source data

Previous analyses of mutational signatures in pediatric cancers, including our own published analysis^2^, have identified a substantial proportion (54% of the whole cohort) of Signature.3 (COSMIC v.2), mostly in tumors without any HR pathway gene defects^2^. However, the current analysis with COSMIC v.3 signatures identified only a small fraction (2.29% of the whole cohort) of tumors with SBS3 signature activity (Fig. 4a,b). This marked difference is most likely the result of previous Signature.3 mutations being attributed to ‘flat’ signatures (for example, SBS5 and SBS40) of the updated and refined COSMIC v.3 mutational signatures. In addition, there is a difference in the approach compared with the initial signature analysis, as we assume SBS1 and SBS5 as background signatures and add SBS3 only if it improves the cosine similarity by at least 0.02. However, none of these tumors with SBS3 showed the associated ID6 signature activity, represented by a high fraction of long deletions at MH regions (Fig. 4b). This lack of ID6 prompted us to test whether our current variant-calling pipeline could be penalizing deletions at microhomologies by assigning low confidence, for example, or whether pediatric cancers in general have very low numbers of MH-associated deletions as an inherent property. To test this further, we whole-genome sequenced five tumors from the INFORM registry (https://www.dkfz.de/en/inform/index.html; INF-WGS sample set)^33^ with known (likely) pathogenic germline mutations in *BRCA1/2* based on ClinVar^46^. Among these, INF_R_1076 had a *BRCA2* pathogenic homozygous mutation and INF_R_025 a *BRCA2* compound heterozygous mutation (both patients also had a clear phenotype, that is, Fanconi anemia), while the remaining tumors had heterozygous (likely) pathogenic *BRCA1/2* mutations (Fig. 4c) without any second hit in the tumor. Mutational signature analysis of these five pediatric tumors revealed that only the tumor with pathogenic homozygous *BRCA2* mutation, that is, biallelic inactivation, showed SBS3 and ID6 signature activity (Fig. 4c and Supplementary Table 14). In addition, we analyzed 22 whole-genome-sequenced adult tumors from the NCT_MASTER precision oncology program^47^ (https://www.nct-heidelberg.de/master) with respect to ID signatures, for which somatic mutations were called with the same DKFZ inhouse pipeline. Among these, half of the tumors (*n* = 11) had known *BRCA1/2* deficiency and showed clear ID6 activity (Extended Data Fig. 8a and Supplementary Table 15), as expected. These results indicate that our variant-calling pipeline does not systematically miss the deletions at MH regions and confirm the importance of biallelic *BRCA1/2* inactivation for the presence of predictive HR defect-associated mutational signatures, that is, SBS3 and ID6.

Next, we utilized classifer of homologous recombination deficiency (CHORD)^48^, a random-forest-based classifier of tumors into *BRCA1* or *BRCA2* HR repair deficiency (HRD) phenotypes given a tumor’s somatic mutational catalog and genomic instability information. We systematically applied CHORD to PPC-WGS and *n* = 5 INF-WGS tumors. Among INFORM tumors, only the single tumor with a *BRCA2* pathogenic homozygous mutation (INF_R_1076) has been identified as *BRCA2* HRD phenotype with high probability (0.82) (Fig. 4d). This tumor also showed activity of SBS3 and ID6 from mutational signature analysis and a low expression of the *BRCA2* gene (Extended Data Fig. 8d). The remaining tumors from INFORM or PPC-WGS (Fig. 4d,e) showed very low probability of being either of the *BRCA1* or *BRCA2* HRD phenotype, suggesting the importance of inactivating *BRCA1/2* biallelic mutations and a combination of SBS3 and ID6 mutational signatures activity as potential biomarker for the so-called BRCAness phenotype.

Next, we sought to understand whether a proportion of long deletions (>5 bp) at microhomologies (MH) are present in pediatric tumors, albeit at very low levels. To understand how MH-associated deletions are represented in ID6 positive adult tumors, we divided the PCAWG whole-genome tumors (*n* = 2,602) into different categories depending on the presence of SBS3 and ID6. Then, we compared the proportion of MH-associated deletions (as a fraction of all deletions) of tumors in these categories with our pediatric cancer cohort. This analysis revealed that pediatric tumors overall have very few MH-associated deletions compared with adult tumors and a significantly lower fraction compared with ID6 positive tumors (Fig. 4f).

**Extended Data Fig. 9:**
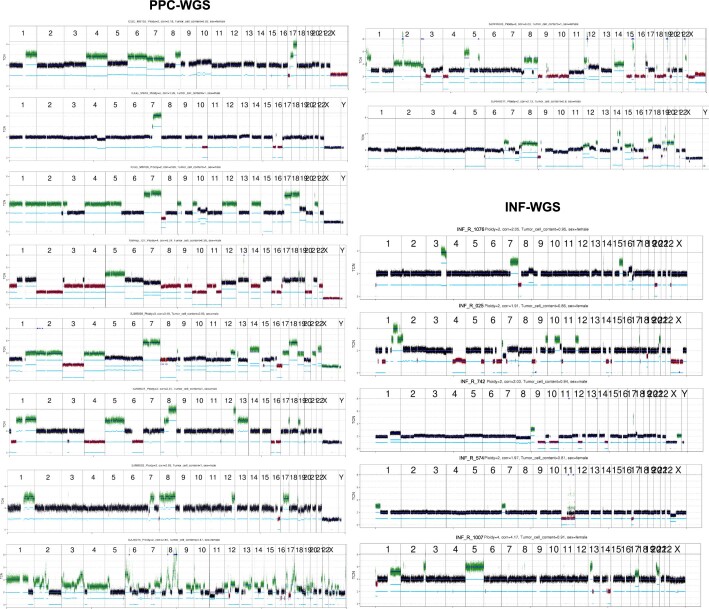
Representative genome-wide copy-number profiles of SBS3 positive tumors from the PPC-WGS cohort and n = 5 INFORM tumors. TCN = Tumor Copy Number. Red indicates loss of genomic material, green indicates gains. Plots were generated using the ACEseq tool. Source data

Among the tumors with SBS3 signature activity in our cohort, 44% (8 of 18) belong to Group3 subgroup of medulloblastoma (MB). MBs are known to follow a linear increase of somatic mutation burden with age^49^. Presumably, mutations in tumors that do not follow such a correlation could be contributed by mutational processes other than the ubiquitous clock-like signatures. In this analysis, we identified only one tumor (SJMB008) with an active SBS3 signature as an outlier of age versus mutation burden correlation, suggesting that most of the other SBS3-positive Group3 MBs follow the typical linear increase in mutation burden with age (Extended Data Fig. 8b), and, therefore, the possibility of misassigning mutations from clock-like signatures (especially SBS5 and SBS40) to SBS3 cannot be excluded. Next, we compared the expression of HR pathway genes such as *BRCA1/2* and *PALB2* between SBS3-positive and -negative tumors in Group3 MBs, since there could be other mechanisms to inactivate the expression of these genes. We have not observed any difference in terms of expression of these genes between SBS3-positive and -negative tumors (Extended Data Fig. 8c). Furthermore, the genome of these SBS3-positive tumors was relatively stable, with very few outliers (Extended Data Fig. 9).

### Signature.P1 similarity to COSMIC v.3 signatures

**Fig. 5:**
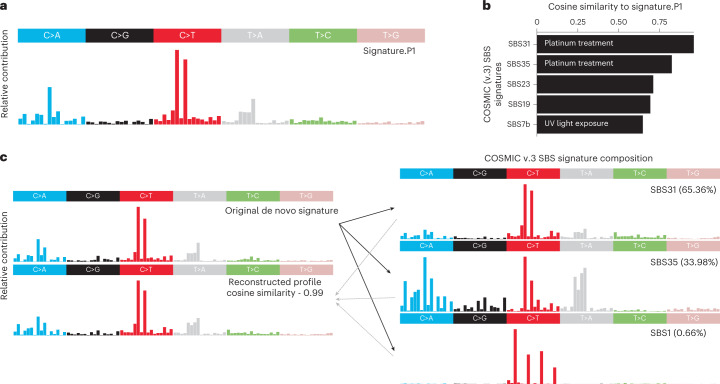
Similarity between Signature.P1 and SBS reference signatures. **a**, SBS profile of Signature.P1 identified in Gröbner et al.^2^. **b**, Cosine similarity between Signature.P1 and COSMIC v.3 SBS signatures. **c**, Decomposition of de novo profile, which is a mixture of SBS31 and SBS35. Source data

Previous mutational signature analysis of a subset of this cohort based on COSMIC v.2 reference signatures identified a new substitution signature, called Signature P1, which featured elevated mutations of C>T in the context of CCC/CCT^2^ (Fig. 5a). Signature.P1 was active in the pediatric brain tumors atypical teratoid/rhabdoid tumors (ATRT) and ependymoma. In the current analysis, the Signature.P1 profile was compared with all identified COSMIC v.3 SBS signatures and a high similarity was observed with SBS31 and SBS35 (Fig. 5b), both of which are the result of DNA damage caused by platinum treatment. In the present analysis with decomposition of de novo signature into COSMIC v.3 signatures, SBS31 and SBS35 were found to be present as a mixture in the Signature.P1 profile (Fig. 5c). This platinum treatment signature activity was identified in ATRT, ependymoma, embryonal tumor with multilayered rosettes (ETMR), and pediatric renal cell carcinoma (RCC) (Fig. 2b and Supplementary Table 6). Furthermore, Lambo et al.^50^ have suggested that most acquired mutations in relapsed ETMRs is induced by cisplatin treatment, resembling signature.P1, since the composition of single-nucleotide variants changed after treatment, but remained similar in subsequent relapses of ETMR.

These results suggest that Signature.P1 is not, as previously hypothesized, a pediatric specific mutational signature, but rather a treatment-associated signature identified in a small fraction of tumors that were annotated as treatment naïve. These patients had probably been treated before genomic analysis and, at least for one ATRT tumor (H049-JVCT; high SBS35 activity; Supplementary Table 6), we could follow up with the sample source, which confirmed that this tumor was a recurrence and not a primary tumor.

## Discussion

In this study, we analyzed 785 whole-genome-sequenced tumor-normal pairs from 27 different molecularly defined entities of childhood cancers to identify and refine the mutational signatures of underlying mutational processes. We examined SBS and small ID signatures in depth and showed that a relatively small number of mutational processes operate in pediatric cancers compared with the typical tumors of adulthood. Among the identified 29 SBS and ten ID signatures, etiologies for more than half of these signatures have been described either by experimental approaches or association analyses in previous studies. In this cohort, a large fraction of SBS (45.4%) and ID (93.2%) mutations across multiple cancer types were attributed to clock-like substitution signatures SBS1 and SBS5, as well as to clock-like indel signatures ID1 and ID2. Signatures such as SBS8 are relatively frequent across cancer entities, and recent evidence suggests this signature is due to DNA damage induced by late replication errors^34^. Other signatures without known etiology were identified in only a small fraction of tumors from specific cancer types such as pilocytic astrocytoma (namely, SBS12 and SBS23) and Burkitt lymphoma (namely, SBS17a/b). However, we did not identify any genomic alterations common to these tumors.

The UV exposure signature SBS7a/b, frequent in adult skin melanomas, was identified in B-ALL hypodiploid tumors in our cohort, with similar transcriptional strand asymmetry as observed in adult tumors. Previous studies reported this mutational signature also in several pediatric ALL patients^3^. A recent study on the genomic landscape of ALL^51^ analyzed in depth whether SBS7 is caused by UV itself or whether it is a biochemical mimic, suggesting that SBS7 is indeed UV-induced in ALL. First, they observed SBS7 primarily in ALL patients of European ancestry, but not in those of African descent. Second, the highest incidence of ALL occurs in patients of European ancestry. Third, extensive chemical profiling has not yet revealed biochemical causes of SBS7 other than UV light.

In our cross-cohort analysis, mutations attributed to SBS8 were significantly higher in tumors affected by chromothripsis. Furthermore, general genomic instability was highly correlated with SBS40 activity, which may indicate that mechanisms involved in genomic rearrangements contribute to the mutational processes underlying SBS8 and SBS40. This is in line with the findings by Gröbner et al.^2^, in which they reported that SBS3, SBS8 and SBS13 were more pronounced in cancer types with higher genomic instability; however, SBS40 was not yet described as a COSMIC signature back then and, as we described here, SBS3 activity in pediatric cancers is rare and rather due to misassigned SBS40. These hypothesis-driven association analyses were based mainly on previous evidence and findings from our 2018 pediatric pan-cancer study^2^, and do not present an unbiased association analysis with all possible clinical and genomic features. Therefore, future studies should include a systematic analysis of all clinical and genomic features and their association with mutational signatures.

Signature.P1 in Gröbner et al.^2^, initially speculated to represent a new SBS signature in pediatric tumors, has been refined in our analysis and observed to contain a mixture of SBS31 and SBS35—two platinum treatment-associated signatures. In addition, Lambo et al.^50^, have suggested that most acquired mutations in relapsed ETMRs are induced by cisplatin treatment, which results in a mutational pattern highly similar to Signature.P1.

In pediatric leukemias, we identified the indel signature that we termed IDN, not yet reported elsewhere, characterized by long, insertions of predominantly more than 5 bp, outside of repeat units, affecting mainly intronic and intergenic regions, but also coding exons of known cancer genes, including *NOTCH1*. Since detection of long insertion indels is difficult with standard short-read sequencing data and computational approaches, it is not unlikely that even longer (and potentially even higher numbers of) insertions affect these leukemia genomes, but were missed with the current indel calling procedures^52^. Since only leukemias were found to be affected by the IDN signature, that is, B-ALL, T-ALL and AML, mutagenic processes intrinsic to myeloid and lymphoid cells might explain the underlying etiology. Mutational signature analysis for substitutions was performed in a recent study of the genomic landscape of relapsed ALL, but not for indels^53^. In the recently published large-scale pan-cancer mutational signature analyses from Alexandrov et al.^14^, 95 chronic lymphocytic leukemias (CLL) from adult patients were included, but no new indel signature was reported for these, whereas Degasperi et al.^54^ studied only substitution mutational signatures. Therefore, it remains to be seen whether indel signature IDN affects leukemias in general or whether it is restricted to the pediatric age group.

Previous mutational signature analyses identified Signature3 (equivalent to the current SBS3) along with other features, with or without discernable alterations in *BRCA1/2* genes, as a potential biomarker for HRD and platinum plus PARPi-based treatment response^55,56^. In pediatric cancers, germline predisposition or somatic alterations in *BRCA1/2* genes are infrequent^2,3^ and only a small fraction (2.29%) of tumors showed SBS3 activity in this analysis, but without the complementing indel signature ID6. MH-associated deletions, a strong feature of ID6, were significantly rarer in pediatric than in adult cancers. A plausible explanation for this observation may be that the total somatic mutation burden of childhood cancers is very low^2^ and the likelihood of mutations occurring at MH sequences will therefore also be small. Furthermore, we do not discount the possibility of misassigning SBS5 and SBS40 signature mutations to SBS3. In addition, a recent study suggests that SBS3 is probably not as specific as previously believed and that the identification of HRD should rely on multiple orthogonal mutational signatures, not only on SBS3 activity^48^. To validate the biomarker efficacy of SBS3 to predict PARP inhibitor treatment response in pediatric cancers, experiments involving patient-derived xenograft models of pediatric tumors are currently ongoing (for example, in the ‘BRCAddict’ project, https://www.transcanfp7.eu/index.php/abstract/brcaddict.html). Recent studies focusing on Ewing sarcoma^57^ and ETMR brain tumors^50^ identified the presence of R-loops, DNA–RNA hybrid structures, to be correlated with PARP inhibitor response. In the future, mutational signature SBS3 combined with other features such as indel signature ID6 and/or R-loops should be investigated in preclinical models to asses PARP inhibitor response and to define optimized biomarkers.

In summary, although some rare cancer entities are under-represented in this cohort, we believe this analysis of the mutational signature repertoire in 27 childhood cancers provides a valuable resource for further understanding of tumor biology and aids future research in defining biomarkers of treatment response.

## Methods

No statistical methods were used to predetermine sample sizes but our sample sizes are similar to those reported in previous publications^2,14^. The experiments were not randomized and investigators were not blinded to allocation during experiments and outcome assessment. Further information on research design is available in the Nature Research Reporting Summary linked to this article.

### Samples

There was no active patient recruitment for the present study; instead, this was a reanalysis of existing datasets and clinical and genomics data were obtained as follows. The pediatric cancer WGS cohort analyzed in this study is a compilation of published individual sequencing datasets from various sources: the International Cancer Genome Consortium (ICGC)—Pedbrain Tumor and MMML-seq as well as Ewing Sarcoma (http://www.icgc.org), the German Cancer Consortium (DKTK) (https://dktk.dkfz.de/en/home), the Pediatric Cancer Genome Project (PCGP) (http://explore.pediatriccancergenomeproject.org/), the Heidelberg Institute for Personalized Oncology (HIPO) (http://www.dkfz.de/en/hipo), the Individualized Therapy For Relapsed Malignancies in Childhood (INFORM) registry (www.dkfz.de/en/inform) and other previously published datasets used in Gröbner at al.^2^.

WGS data for primary tumor samples from 149 pediatric patients were obtained from St. Jude Cloud^58^. St. Jude Cloud data included PCGP, Genomes 4 Kids (G4K), Real-time Clinical Genomics (RTCG) and Childhood Solid Tumor Network (CSTN).

For all included tumors, matched germline control tissue was available. All centers have approved data access and informed consent had been obtained from all patients by the respective centers.

### WGS data, alignment and variant calling

A subset of the WGS data analyzed in this study was collected from Gröbner et al.^2^ and remaining data were collected from St. Judes pediatric cancer genome project^59–61^ (https://www.stjude.org/research/translational-innovation/pediatric-cancer-genome-project.html). Supplementary Table 1 contains the information on which tumors were downloaded from St Judes cloud. Briefly, FASTQ data were aligned to reference genome GRCh37/hg19 with BWA-MEM (v.0.7.8)^62^. SBSs were called using an updated Samtools^63^-based DKFZ inhouse pipeline (v.0.1.19) and indels were called using Platypus^64^ (v.0.8.1.1). In addition, variant calling was also performed with mutect2 (ref. ^21^) (v.GATK/4.0.8.1) with the standard recommended parameters for tumor-normal pairs. Finally, somatic variants from both pipelines were overlapped with ‘bedtools intersect’^65^ (bedtools v.2.30.0; bedtools intersect -u -a vcfFileDkfz -b vcfFileMutect2) and created a consensus variant call set for each tumor. For mutational signature analysis in this manuscript, only consensus somatic variants have been used.

### Structural variants and copy-number profiles

Structural variants per tumor were identified as described previously using the DELLY^66^ ICGC pan-cancer analysis workflow (https://github.com/ICGC-TCGA-PanCancer/pcawg_delly_workflow) or SOPHIA (https://bitbucket.org/utoprak/sophia/src/master). Copy-numbers were also estimated using ACEseq (allele-specific copy-number estimation from sequencing), based on binned tumor-control coverage ratio and B-allele frequencies (BAF)^67^.

### Somatic mutation frequencies

SBS and small ID mutation burden was calculated as the total number of mutations identified per megabase of the genome. For WGS data, the total number of mutations was divided by 2,800 (effective human genome size in megabases that can be assessed by WGS).

### Generation of mutational catalogs

Somatic mutational catalogs of SBS and ID were generated using SigProfilerMatrixGenerator. We generated SBS96, SBS1536, ID83 catalogs for each cancer type and subsequently used these matrices as input for mutational signature extraction.

### Extraction of mutational signatures using SigProfilerExtractor

SigProfilerExtractor is a Python-based tool that employs NMF to extract optimal number of mutational signatures in a given cohort of tumors, and attributes mutations to each signature within each tumor. For a given mutational catalog M, we ran factorization of M with different ranks ranging from *k* = 1 to *k* = 10. For each value of *k*, we performed 500 independent non-negative matrix factorizations of M, resulting in 500 different matrices S, reflecting de novo signatures and additional 500 matrices A, reflecting activities of de novo signatures in each tumor. Initial S and A matrices for the first step of factorization were initialized randomly and each factorization was performed with a custom implementation of the multiplicative update algorithm^68^, minimizing the objective function Kullback–Leibler divergence. We used default parameters for NMF multiplicative update steps (minimum 10,000 and maximum 1,000,000) and algorithm convergence tolerance 10 × 10^–15^. SigProfilerExtractor then applies a custom clustering algorithm for each value of *k* and produces consensus mutational signatures, reconstruction error describing the original input matrix M and finally stability value for each identified consensus mutational signatures. The optimal solution *k* is selected based on their average stability (≥ 0.80) with no individual de novo signature having stability less than 0.2.

Since de novo mutational signatures can be a mixture of multiple signatures, each de novo extracted signature was further decomposed into a set of known COSMIC (v.3) (https://cancer.sanger.ac.uk/signatures/) signatures using non-negative least squares (NNLS) algorithm requiring a minimum cosine similarity of 0.85 for all reconstructed signatures with a set of signature addition and removal steps as described^69^. The mathematical selection optimal *k* is not always accurate^70^, hence we visually inspected optimal solution selection plots and decomposition plots and manually adjusted the selected *k* by ± 1 in 5.4% of the cases (3 of 55 extractions).

### Extraction of mutational signatures using SignatureAnalyzer

SignatureAnalyzer depends on a Bayesian variant of NMF that infers the optimal number of signatures via automatic relevance determination technique (ARD-NMF). The same methodology has been implemented in producing reference catalog of mutational signatures as part of PCAWG efforts^14^. To extract mutational signatures, we generated SBS1536 (considering two bases before and after the mutated base) and ID83 catalog as previously described, and produced a COMPOSITE spectra combining SBS1536 + ID83 catalogs^14^. We used these COMPOSITE spectra containing 1,619 features as input to SignatureAnalyzer Python version to extract SBS signatures, with default recommended parameters for mutational signature extraction. The de novo signatures were then collapsed into SBS96 features to generate SBS de novo signatures. A separate extraction with ID83 mutational spectra was carried out to extract ID de novo signatures.

As with SigProfilerExtractor analysis, we applied the same procedure to decompose these de novo signatures to a set of known COSMIC v.3 signatures, requiring a minimum cosine similarity greater than 0.8 for the reconstructed signature. We then applied supervised ARD-NMF to assign mutations to each signature within each tumor using Poisson objective function. Due to the overfitting characteristics of SigAnalyzer attribution, we considered a signature if it contributes at least 5% of mutations to the overall mutation burden in a tumor.

**Extended Data Fig. 10:**
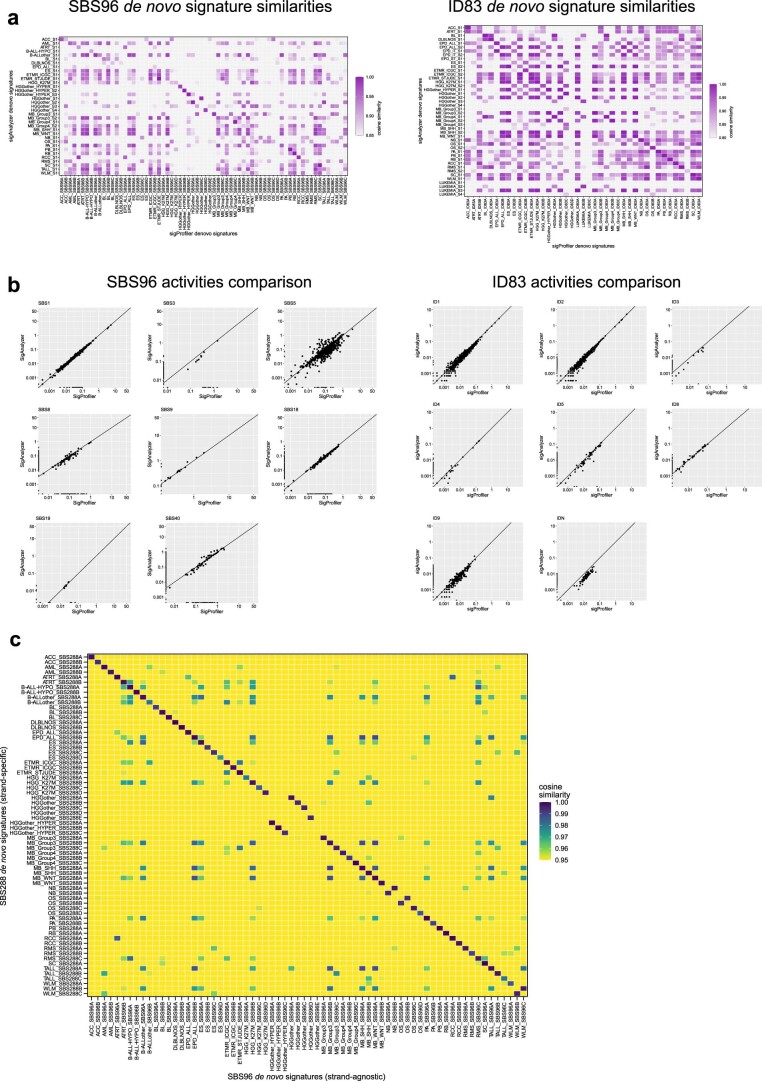
Comparison of *de novo* profiles and activities estimated by SigProfiler and SigAnalyzer. a) Comparison of SBS and ID *de novo* signature profiles extracted by SigProfiler and SigAnalyzer b) Comparison of SBS and ID signature activities estimated by SigProfiler and SigAnalyzer of n = 785 tumors c) Comparison of *de novo* profiles of strand-specific (SBS288) with strand-agnostic (SBS96) SBS signatures of n = 785 tumors. Source data

The results of both SigProfilerExtractor and SigAnalyzer are visualized according to the signature PCAWG mutational publication^14^. In the visualizations presented in Fig. 2b and Extended Data Fig. 3, we considered only tumors that show at least 20 mutations. However, in the supplementary tables and Extended Data Figs. 4 and 5, the whole cohort has been presented. A comparison of de novo profiles and activities from both methods is presented in Extended Data Fig. 10a,b. In addition, strand-specific signatures were extracted with SigProfiler and compared with strand-agnostic SBS96 signature profiles to check the consistency of the extracted de novo profiles. The signature extraction procedure was similar to SBS96 analysis, except that SigProfilerExtractor was run in SBS288 mode. The resulting SBS288 de novo profiles were collapsed into SBS96 signatures by summing the transcribed and untranscribed trinucleotide probabilities. The resulting de novo profiles were compared with the initial set of SBS96 signatures extracted using cosine similarity measure and visualized in a heatmap (Extended Data Fig. 10c).

### Extraction of indel signatures across leukemia cohort using HDP

For the sake of robustness of the identified new ID signature we termed IDN, we implemented the non-NMF-based algorithm HDP across leukemia tumors in our cohort. The algorithm was conditioned on a set of indel signatures that are commonly identified (ID1, ID2, ID5, ID8 and ID9). This allows simultaneous discovery of new signatures and also the matching of extracted signatures to known signatures, if the cosine similarity is ≥0.9. The details of the algorithm have been described^29^.

### Attribution of mutational signatures from *n* = 5 INFORM WGS tumors

Since the additionally sequenced INFORM tumors belong to distinct entities, instead of extraction we performed attribution of mutations to COSMIC v.3 reference SBS and ID signatures using deConstrucSigs^71^. This approach determines the linear combination of predefined signatures (that is, COSMIC v.3 signatures) that accurately reconstruct the given tumor profile using a multiple linear regression model.

### Mappability assessment of indels

To estimate the mappability of indels assigned to each of the ID signatures, we first defined indels in each tumor due to each signature from SigProfilerExtractor results. We then used the hg19 100 basepair mappability tracks (https://zenodo.org/record/5521424#.Y5M0oezMKb8) to estimate the mappability score for each of the indels in each tumor and visualized as a distribution plot. These hg19 mappability tracks were generated using Genome Multitool (GEM) program as described in Derrien et al.^72^.

### CHORD analysis of PPC-WGS and INF-WGS

To identify whether a given tumor has HRD, we implemented the CHORD algorithm on PPC and INFORM WGS data. CHORD is a random-forest-based classifier trained on more than 5,000 whole-genome-sequenced tumors to robustly identify tumors with *BRCA1* or *BRCA2* type HRD phenotype. CHORD requires SBS and small ID mutations and the length of each structural variant in each tumor and results in a probability value for *BRCA1* and *BRCA2* type HRD for each tumor. We prepared the required files from consensus variant calls and DELLY or SOPHIA structural variants. We considered a tumor is HRD phenotype if the CHORD-produced probability value is greater than 0.5 for either *BRCA1* or *BRCA2* type.

### Germline mutation analysis

Identifying mutations in genes predisposing to cancer was performed as described in the pan-cancer study by Groebner et al.^2^. https://www.nature.com/articles/nature25480#Sec9 To identify germline variants with a high likelihood of being implicated in cancer development, we investigated 162 candidate genes adapted from https://pubmed.ncbi.nlm.nih.gov/27479119/ (110 genes regarded as following a dominant inheritance pattern and 52 genes with recessive inheritance).

Germline single-nucleotide variants and indels were subjected to a stepwise filtering approach to eventually classify them into five categories: benign, likely benign, uncertain significance, likely pathogenic and pathogenic. First, variants reported in both the 1000 Genomes (release November 2010) and dbSNP (v.141) databases were excluded. High-quality variant calls were selected by including only positions with ≥15× coverage, a germline allele frequency of ≥0.2 and a phred-based quality score of ≥10. Variants with a population frequency ≥0.01 reported in additional common databases (esp6500siv2, X1000g2015 and exac03 included in ANNOVAR (http://annovar.openbioinformatics.org)) or with ClinVar (ftp://ftp.ncbi.nlm.nih.gov/pub/clinvar/) annotations of ‘benign’, ‘likely benign’ or ‘uncertain significance’ were removed.

Furthermore, variants with a phred-scaled CADD score ≥15 (http://cadd.gs.washington.edu/info) and with Mutation Assessor (http://mutationassessor.org/r3/) categories ‘medium’ and ‘high’, or no available annotation, were included. Variants with a dbSNP classification of ‘precious’ were not subject to these two filtering steps. As indel calling is more prone to alignment and calling errors, potentially deleterious indels were manually investigated for artefacts. For recessive tumor genes, variants were included only with an allele frequency of one or with two compound heterozygous mutations of the same gene in the same patient. Every variant was then checked manually and scored by the use of varied, mainly gene-specific online databases (http://p53.iarc.fr/, http://www.lovd.nl/3.0/home, https://www.ncbi.nlm.nih.gov/clinvar/ and others). Only probable pathogenic and pathogenic mutations were considered as cancer-relevant. Finally, *TP53* and *BRCA1/2* gene mutations were considered for signature association analysis.

### Association of signature activity with *TP53*, chromothripsis and genomic instability

We determined the association of signature activity and TP53 or chromothripsis using a linear regression model with cancer type and age as covariates. Signatures that are identified in at least five tumors and three different cancer types were considered for association analysis. We used the lm function in R with the formula lm(exposure ~ TP53 status + cancer type + age). The resulting *P* values were adjusted using the Benjamini–Hochberg method. The associations with a positive coefficient for the given independent variable of interest from linear model and adjusted *P* value <0.05 were considered as significant associations.

Genomic instability is quantified as the sum of the different structural variants identified in a tumor. For the association analysis of mutational signature exposure and genomic instability, we performed correlation analysis of these two features. If the *P* value <0.05 and correlation is >0.2, we considered it a significant association.

### Strand asymmetry analysis of mutational signatures

We used SigProfilerMatrixGenerator to evaluate if a mutation (either SBS or ID) occurs on a transcribed or untranscribed strand and in a lagging or leading region of the genome as described^39,73^. Strand asymmetry analysis was performed using SigProgilerTopography^39^. Briefly, the ratio of transcribed versus untranscribed and lagging versus leading strands is calculated and compared with the respective ratios of the simulated dataset. Each cancer type was evaluated separately with 100 simulations. If the ratios were significantly different in observed data compared with simulated data (Fischer exact test with Benjamini–Hochberg adjusted *P* value <0.05), then only a signature is reported to exhibit strand asymmetry.

### Chromothripsis detection

For all the tumors overlapping with the Gröbner et al.^2^ study, we used the same chromothripsis annotations provided in that study. For the tumors newly added to this study, we used the ShatterSeek R package with default parameters. ShatterSeek was developed as part of the PCAWG chromothripsis analysis study^74^. For all tumors for which shatterSeek detected chromothripsis events, their tumors/normal copy number profiles were inspected visually to confirm.

### Statistics and reproducibility

No statistical methods were used to predetermine sample size. The experiments were not randomized and investigators were not blinded to allocation during experiments and outcome assessment. All parameters used in NMF and HDP for signature extraction were described in relevant methods sections. For all the comparison and correlation analyses, exact *P* values were reported in the figures. Unless otherwise specified, *P*_adj_ means adjusted *P* values with Benjamini–Hochberg method. Data distributions were assumed to be normal, but this was not formally tested. Unless otherwise specified, all values were included, median and interquartile ranges were shown in all boxplots with whiskers extending 1.5× interquartile range.

### Reporting summary

Further information on research design is available in the Nature Portfolio Reporting Summary linked to this article.

## Supplementary information

Reporting Summary

Supplementary Tables Table includes tumor sample annotation, cohort overview and mutational signatures per sample and from different signature extraction methods. Several spreadsheet-based tables combined into a single workbook with multiple tabs.

## Data Availability

The consensus variant calls generated as part of this study for 785 tumors are available on Synapse (https://www.synapse.org/) under the id ‘syn35289647’. The WGS data we had already published in the 2018 pediatric pan-cancer landscape study^2^ comprised the following datasets: DKFZ external data were downloaded from the European Genome-Phenome Archive (EGA; https://www.ebi.ac.uk/ega/home) using the accession numbers EGAD00001000085, EGAD00001000135, EGAD00001000159, EGAD00001000160, EGAD00001000161, EGAD00001000162, EGAD00001000163, EGAD00001000164, EGAD00001000165, EGAD00001000259, EGAD00001000260, EGAD00001000261, EGAD00001000268 and EGAD00001000269; DKFZ internal datasets are related to previous PMIDs 27748748, 27479119, 26923874, 25670083, 25253770, 24972766, 24553142, 25135868, 26632267, 26179511, 24651015, 28726821, 23817572, 25962120, 26294725. WGS data for a subset (*n* = 149) of pediatric tumor samples used for analysis in this study were obtained from St. Jude Cloud^58^ (https://www.stjude.cloud), where the data can be accessed or downloaded. Pediatric Cancer Genome Project (PCGP), Genomes 4 Kids (G4K), Real-time Clinical Genomics (RTCG) and Childhood Solid Tumor Network (CSTN) raw data (BAM files) are available at St. Jude Cloud under controlled access; data access requests are reviewed by the respective Steering Committee. The Ewing Sarcoma WGS data (‘Genomic landscape of Ewing sarcoma (ICGC project)’) access was obtained through the International Cancer Genome Consortium Data Portal at https://dcc.icgc.org/ and was downloaded from EGA using the accession number EGAD00001001051. INFORM data (*n* = 5) used in this study are available from the European Genome Archive, accession number EGAS00001005112. Source data are provided with this paper.

## Source data

Source Data Figs. 1–5 and Extended Data Figs. 1–10 Statistical source data.

## Code availability

SigProfilerMatrixGenerator (v.1.2.4) (https://github.com/AlexandrovLab/SigProfilerMatrixGenerator), SigProfilerExtractor (v.1.1.7) (https://github.com/AlexandrovLab/SigProfilerExtractor), SignatureAnalyzer (v.0.0.8) (https://github.com/getzlab/SignatureAnalyzer), deConstrucSigs (v.1.9.0) (https://github.com/raerose01/deconstructSigs), SigProfilerTopography (v.1.0.63) (https://github.com/AlexandrovLab/SigProfilerTopography), CHORD (v.2.0) (https://github.com/UMCUGenetics/CHORD), shatterSeek (v.1.1) (https://github.com/parklab/ShatterSeek) and code written for this manuscript along with tutorial (https://github.com/KiTZ-Heidelberg/Signatures-Manuscript). All analyses were performed either with Python (v.3.7.3) or R statistical programming (v.4.0.5).
