# Supplementary material for: Comprehensive analysis of mutational signatures reveals distinct patterns and molecular processes across 27 pediatric cancers
Source: Nat Cancer. 2023 Jan 26;4(2):276–89. doi: 10.1038/s43018-022-00509-4 (PMC9970869; doi:10.1038/s43018-022-00509-4)
Supplement: Supplementary file 1 — Reporting Summary [file 43018_2022_509_MOESM1_ESM.pdf]

nature portfolio

Corresponding author(s): Stefan M. Pfister, Natalie Jäger

Last updated by author(s): Dec 10, 2022

Reporting Summary

Nature Portfolio wishes to improve the reproducibility of the work that we publish. This form provides structure for consistency and transparency in reporting. For further information on Nature Portfolio policies, see our Editorial Policies and the Editorial Policy Checklist.

Statistics

For all statistical analyses, confirm that the following items are present in the figure legend, table legend, main text, or Methods section.

n/a	Confirmed	
☐	☒	The exact sample size (n) for each experimental group/condition, given as a discrete number and unit of measurement
☐	☒	A statement on whether measurements were taken from distinct samples or whether the same sample was measured repeatedly
☐	☒	The statistical test(s) used AND whether they are one- or two-sided<br>*Only common tests should be described solely by name; describe more complex techniques in the Methods section.*
☐	☒	A description of all covariates tested
☐	☒	A description of any assumptions or corrections, such as tests of normality and adjustment for multiple comparisons
☐	☒	A full description of the statistical parameters including central tendency (e.g. means) or other basic estimates (e.g. regression coefficient) AND variation (e.g. standard deviation) or associated estimates of uncertainty (e.g. confidence intervals)
☐	☒	For null hypothesis testing, the test statistic (e.g. F, t, r) with confidence intervals, effect sizes, degrees of freedom and P value noted<br>*Give P values as exact values whenever suitable.*
☒	☐	For Bayesian analysis, information on the choice of priors and Markov chain Monte Carlo settings
☒	☐	For hierarchical and complex designs, identification of the appropriate level for tests and full reporting of outcomes
☐	☒	Estimates of effect sizes (e.g. Cohen's d, Pearson's r), indicating how they were calculated

Our web collection on statistics for biologists contains articles on many of the points above.

Software and code

Policy information about availability of computer code

Data collection: As this study was a re-analysis of available published data, no additional software was used for data collection.

Data analysis:
Statistical analysis and code availability:
SigProfilerMatrixGenerator (v 1.2.4) (https://github.com/AlexandrovLab/SigProfilerMatrixGenerator),
SigProfilerExtractor (v 1.1.7) (https://github.com/AlexandrovLab/SigProfilerExtractor),
SignatureAnalyzer (v 0.0.8) (https://github.com/getzlab/SignatureAnalyzer),
deConstrucSigs (v 1.9.0) (https://github.com/raerose01/deconstructSigs),
SigProfilerTopography (v 1.0.63) (https://github.com/AlexandrovLab/SigProfilerTopography),
CHORD (v 2.0) (https://github.com/UMCUGenetics/CHORD),
shatterSeek (v 1.1) (https://github.com/parklab/ShatterSeek),
code written for this manuscript along with tutorial (https://github.com/KiTZ-Heidelberg/Signatures-Manuscript).

All the analysis has been performed either with python (v 3.7.3) and R statistical programming (v 4.0.5).

For manuscripts utilizing custom algorithms or software that are central to the research but not yet described in published literature, software must be made available to editors and reviewers. We strongly encourage code deposition in a community repository (e.g. GitHub). See the Nature Portfolio guidelines for submitting code & software for further information.

Data

Policy information about availability of data

All manuscripts must include a data availability statement. This statement should provide the following information, where applicable:

- Accession codes, unique identifiers, or web links for publicly available datasets
- A description of any restrictions on data availability
- For clinical datasets or third party data, please ensure that the statement adheres to our policy

The consensus variant calls generated as part of this study for 785 tumors are available on Synapse (https://www.synapse.org/) under the id "syn35289647".

The WGS data we had already published in the 2018 pediatric pan-cancer landscape study (Groebner et al. Nature 2018) comprised the following data sets: DKFZ external data were downloaded from the European Genome-Phenome Archive (EGA; https://www.ebi.ac.uk/ega/home) using the accession numbers EGAD00001000085, EGAD00001000135, EGAD00001000159, EGAD00001000160, EGAD00001000161, EGAD00001000162, EGAD00001000163, EGAD00001000164, EGAD00001000165, EGAD00001000259, EGAD00001000260, EGAD00001000261, EGAD00001000268, and EGAD00001000269; DKFZ internal datasets are related to previous PMIDs 27748748, 27479119, 26923874, 25670083, 25253770, 24972766, 24553142, 25135868, 26632267, 26179511, 24651015, 28726821, 23817572, 25962120, 26294725.

Whole genome sequencing data for a subset (n=149) of pediatric tumor samples used for analysis in this study were obtained from St. Jude Cloud (https://www.stjude.cloud), from where the data can be accessed or downloaded. Pediatric Cancer Genome Project (PCGP), Genomes 4 Kids (G4K), Real-time Clinical Genomics (RTCG), and Childhood Solid Tumor Network (CSTN) raw data (BAM files) are available at St. Jude Cloud under controlled access; data access requests are reviewed by the respective Steering Committee.

The Ewing Sarcoma WGS data ("Genomic landscape of Ewing sarcoma (ICGC project)") access was obtained through the International Cancer Genome Consortium Data Portal at https://dcc.icgc.org/ and was downloaded from EGA using the accession number EGAD00001001051. INFORM data (n=5) used in this study are available from the European Genome Archive, accession number EGAS00001005112.
Source data are provided with this paper.

Human research participants

Policy information about studies involving human research participants and Sex and Gender in Research.

Reporting on sex and gender

There was no active patient recruitment for the present study, instead this was a reanalysis of existing and published datasets. Sex and gender were not considered in the study design. Sex of cancer patients was used as reported by the clinical data provided by the respective centers that offered the genomics data download, it is reported in Suppl. Table 1. No information on sex was available for 150 of the 785 tumor samples (19%). Sex- and gender based analysis was not performed, since focus was on genomic alterations and mutational signatures across cancer types.

Population characteristics

The cohort analysed in this study is a compilation of individual tumor sequencing datasets from various sources (details described in the manuscript and above). The majority (96%) of patients were under 21 years of age (or age unspecified (n=44) but confirmed as age group paediatric), but available data were included for patients up to 50 years.

Recruitment

There was no active patient recruitment for the present study, instead this was a reanalysis of existing and published datasets and clinical and genomics data were obtained as follows.
The pediatric cancer WGS cohort analysed in this study is a compilation of published individual sequencing datasets from various sources: the International Cancer Genome Consortium (ICGC) – Pedbrain Tumor and MMML-seq as well as Ewing Sarcoma (http://www.icgc.org), the German Cancer Consortium (DKTK) (https://dktk.dkfz.de/en/home), the Pediatric Cancer Genome Project (PCGP) (http://explore.pediatriccancergenomeproject.org/), the Heidelberg Institute for Personalized Oncology (HIPO) (http://www.dkfz.de/en/hipo), the Individualized Therapy For Relapsed Malignancies in Childhood (INFORM) registry (www.dkfz.de/en/inform), and other previously published datasets used in Gröbner at al., 2018, Nature.
Whole genome sequencing data for primary tumor samples from 149 pediatric patients were obtained from St. Jude Cloud. St. Jude Cloud data included Pediatric Cancer Genome Project (PCGP), Genomes 4 Kids (G4K), Real-time Clinical Genomics (RTCG), and Childhood Solid Tumor Network (CSTN).

Ethics oversight

All centres have approved data access and informed consent had been obtained from all patients by the respective centers. All patient material was collected after receiving written informed consent, in accordance with the respective Institutional Review Board guidelines.

Note that full information on the approval of the study protocol must also be provided in the manuscript.

Field-specific reporting

Please select the one below that is the best fit for your research. If you are not sure, read the appropriate sections before making your selection.

☒ Life sciences ☐ Behavioural & social sciences ☐ Ecological, evolutionary & environmental sciences

For a reference copy of the document with all sections, see nature.com/documents/nr-reporting-summary-flat.pdf

Life sciences study design

All studies must disclose on these points even when the disclosure is negative.

Sample size	No statistical methods were used to pre-determine sample sizes, but our sample sizes are similar to those reported in previous publications on pan-cancer mutational signature analyses (Groebner et al., Nature, 2018; Alexandrov et al., 2020). This was an exploratory analysis using available published WGS data sets for pediatric cancers.
Data exclusions	No data were excluded from the analysis.
Replication	No additional, new pediatric tumor WGS data sets were used to replicate our findings on mutational signatures beyond our cohort of n=785+5 samples.
Randomization	The experiments were not randomized.
Blinding	Investigators were not blinded to allocation during experiments and outcome assessment, as blinding was not relevant to this computational re-analysis of the mutational signatures landscape of pediatric cancers. No clinical trials or new patient recruitment was part of this study.

Reporting for specific materials, systems and methods

We require information from authors about some types of materials, experimental systems and methods used in many studies. Here, indicate whether each material, system or method listed is relevant to your study. If you are not sure if a list item applies to your research, read the appropriate section before selecting a response.

Materials & experimental systems

n/a	Involved in the study	
☒	☐	Antibodies
☒	☐	Eukaryotic cell lines
☒	☐	Palaeontology and archaeology
☒	☐	Animals and other organisms
☒	☐	Clinical data
☒	☐	Dual use research of concern

Methods

n/a	Involved in the study	
☒	☐	ChIP-seq
☒	☐	Flow cytometry
☒	☐	MRI-based neuroimaging

March 2021
